# Anti‐Hyperlipidemic and Anti‐Atherogenic Effect of Citrus Peel Pectin Against Cholesterol and Cholic Acid Induced Hyperlipidemia in Sprague Dawley Rats

**DOI:** 10.1002/fsn3.70274

**Published:** 2025-05-28

**Authors:** Hina Rasheed, Ammar B. Altemimi, Roshina Rabail, Sidra Tul Muntaha, Allah Rakha, Usman Haider, Fareha Rasheed, Maham Shehzad, Amin Mousavi Khaneghah, Gholamreza Abdi, Rana Muhammad Aadil

**Affiliations:** ^1^ National Institute of Food Science and Technology University of Agriculture Faisalabad Pakistan; ^2^ Food Science Department, College of Agriculture University of Basrah Basrah Iraq; ^3^ College of Medicine University of Warith Al‐Anbiyaa Karbala Iraq; ^4^ Faculty of Veterinary Science, Institute of Physiology and Pharmacology University of Agriculture Faisalabad Pakistan; ^5^ Department of General Medicine Pakistan Institute of Medical Sciences Islamabad Pakistan; ^6^ Faculty of Biotechnologies (BioTech) ITMO University Saint Petersburg Russia; ^7^ Department of Biotechnology, Persian Gulf Research Institute Persian Gulf University Bushehr Iran

**Keywords:** atherogenic index, citrus peel pectin, hyperlipidemia, LDL, total cholesterol, triglycerides

## Abstract

This study was designed to explore the anti‐hyperlipidemic and anti‐atherogenic effects of citrus peel‐derived pectin. A total of 70 Sprague Dawley rats were categorized into 7 groups with 10 rats in each group. Hyperlipidemia was induced in all groups by adding cholesterol (1.5%) and cholic acid (0.5%) to the normal diet for 15 days. G_0_ was considered a normal control, G_1_ was considered a negative control, G_2_ was considered the positive control treated with the standard drug atorvastatin (10 mg/kg p.o.), G_3_, G_4_, G_5_, and G_6_ were given 3%, 7%, 11%, and 15% of citrus peel pectin, respectively, for 60 days. After the trial completion, biochemical markers like lipid profile, liver and renal function tests, and atherogenic index as well as histopathological analysis of heart, liver, and kidney were performed. The results showed a significant decline in TC, TG, and LDL, while significantly increased serum HDL levels consequently reduced the atherogenic index (*p* < 0.05). The TG levels were positively correlated with the atherogenic index, whereas HDL levels were negatively correlated with the atherogenic index. The outcome further suggested that the treatment with citrus peel pectin (15%) significantly enhanced the antioxidant levels and restored the liver and renal function as evidenced by the histopathological studies. Additionally, the findings revealed a significant reduction in the pathological changes of heart tissue treated with citrus peel pectin at different ratios. The highest reduction was seen in G_6_ (treated with 15% citrus peel pectin). As a result of pectin's safety and considerable anti‐hyperlipidemic, anti‐atherogenic, and antioxidant effects, it could be evaluated as a therapeutic option for hyperlipidemia. Thus, citrus peel pectin can be used as an adjunct functional food in the diet to manage hyperlipidemia and improve atherogenesis.

## Graphical abstract

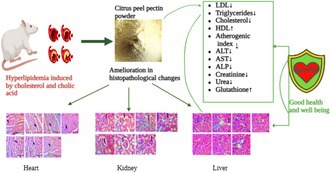

## 1 Introduction

Plant parts or plant extract have been shown to have potential against different diseases, and several studies have proved it (Cissokho et al. 2024; Al‐Nabati et al. 2024; Balgoon and Alghamdi 2024; Rashid et al. 2024). Among these, citrus by‐products, such as peel, which is often considered waste, are a rich source of plant secondary metabolites. Processing industries of citrus produce a large number of wastes each year. Meanwhile, 50% of citrus peel is wasted (Mohamed et al. 2023; Tahir et al. 2023). Citrus peel is low in calories, fat, and sugar and contains flavedo and albedo. It is abundant in fibers, vitamin C, essential oil, polyphenols, and other functional ingredients. Phenolic compounds, including flavanones and polymethoxylated flavones, have been reported to be the main water‐soluble part of citrus peel (M'hiri et al. 2017; Mohamed et al. 2023). Additionally, the peels of citrus fruits are the major source of pectin polysaccharides, which can be utilized for numerous applications in the industry (Tahir et al. 2023).

The importance of pectin has been highlighted because of its beneficial effects on the health of humans (Paesani et al. 2020). Furthermore, it aids in reducing the cardiovascular disease risk factors (Indahsari et al. 2020). Pectin is a heterogeneous substance, the major sources of which are peel, pomace, and pulp (Wilkowska et al. 2019). Pectin's ability to form gel has allowed this biopolymer to be exploited in the drug industry and for the promotion of health. Moreover, it reduces the rate of absorption by restraining food components in the digestive system, which in turn decreases the intake of food (Talab 2015).

On a dry weight basis, a whole fruit comprises 3%–7% pectin compounds, and 0.1%–1.1% on a fresh weight basis (Talab 2015). Moreover, citrus peels contain 20%–30% of pectin, which is light cream or light tan (Ruano et al. 2019). Pectin is obtained from the waste of the fruits like oranges (8.79%–26.55% w/w), grapefruit (20%–30% w/w), and lemons (5.52%–28.57% w/w) peels (El Fihry et al. 2022; Zhao et al. 2021). Researchers are focusing on the processing of such waste that can be processed further to retain their bioactive compounds and use them in the treatment of various diseases (Singh et al. 2020). Globally, citrus waste surpasses about 110–120 million tons per year. Thus, it is necessary to process this waste to prevent serious environmental pollution (Khan et al. 2021). All in all, citrus peel is a good source of pectin (a soluble fiber) with a potential nutraceutical role in the mitigation of cardiovascular disease risk factors.

In recent decades, due to environmental, physiological, lifestyle, biochemical, and metabolic influences, metabolic syndrome has become a major health concern and a threat to the population. The prevalence ranges from 20% to 25% globally with ethnic and gender differences (Lear and Gasevic 2019; Song et al. 2017). Due to sudden shifts in eating habits and lifestyle behaviors, the prevalence of these types of disorders is currently growing in developing countries relative to developed countries (Misra et al. 2018). Hypercholesterolemia is a symptom of lipid metabolism disorders, which is the key risk factor for causing heart disease. Meanwhile, dyslipidemia is an anomaly in lipid levels (Indahsari et al. 2020). Hyperlipidemia is considered a matter of public health, necessitating the urgent need for effective prevention and treatment. Nevertheless, the management of hyperlipidemia remains relatively limited (Wang et al. 2023). Non‐communicable diseases (NCDs), responsible for 43 million deaths in 2021, globally constitute nearly 75% of non‐pandemic‐related deaths. Almost 18 million NCDs occur before the age of 70 years, and 82% are estimated to occur in low‐ and middle‐income countries. Out of these NCDs, cardiovascular diseases (CVDs) contributed to most of the deaths (19 million) in 2021 (WHO 2024). The global prevalence of dyslipidemia was found to be 20%–80% according to a systematic review protocol based on the criteria used (Mohamed‐Yassin et al. 2021). According to the American Heart Association report, high LDL is responsible for about 3.72 million deaths globally in 2021 (Martin et al. 2024). In this regard, the use of medicinal plants is important and is effective for the treatment of different diseases (Bagheri et al. 2024).

Thus, keeping in view the therapeutic potential of citrus peel pectin, the present study was designed to investigate the impact of citrus peel‐derived pectin on the lipid profile, atherogenic index, and physicochemical parameters such as liver function and renal function markers in cholesterol and cholic acid‐induced hyperlipidemic Sprague Dawley rats. This research was expected to reveal the anti‐hyperlipidemic effects of citrus peel pectin, as well as study the relationship between the atherogenic index and lipid profile. Furthermore, we evaluated the histopathological changes in heart, liver, and kidney tissues following the supplementation of citrus peel pectin to identify key cellular and structural alterations that correlate with treatment efficacy and disease progression. Altogether, our work may highlight the important functional role of citrus peel pectin and provide clues for adding the pectin into commercial products.

## 2 Materials and Methods

### 2.1 Chemicals and Reagents

Citrus peel‐derived pectin (galacturonic acid ≥ 74.0%, methoxy groups 6.7%) was purchased from F.A. traders, Faisalabad. Cholesterol, cholic acid, as well as all other chemicals and reagents used in this research, were purchased from Sigma Aldrich St. Louis, USA, and were of analytical grade. The drug Atorvastatin 40 mg oral tablets were purchased from a nearby Care Pharmacy, Jinnah Colony, Faisalabad, Pakistan.

### 2.2 Animals

The research was carried out to test the hypocholesterolemic potential of citrus peel‐derived pectin. The study was conducted for 60 days on Sprague Dawley rats. Seventy rats (8–10‐weeks‐old males with 150–200 g weight) were procured from the National Institute of Health, Islamabad. Rats were placed individually in a controlled environment and were kept in a pathogen‐free environment. The experiments were performed according to the approved ethical guidelines of the “Institutional Biosafety and Bioethics Committee” of the University of Agriculture, Faisalabad, Pakistan (vide No. 5285/ORIC, dated 28‐08‐2024). The rats were acclimatized for 7 days in the animal house, were kept in stainless steel cages, and were provided with a temperature of about 22°C ± 2°C, a 12/12 period of day and night feeding of standard rat diet (Catalog number: 5LF2) specifications: crude protein 14.3%, crude fat 2.5%, carbohydrates 65.2%, sucrose 0.94%, and fiber 3.7%, and free access to tap water was provided all the time (ad libitum). Then rats were divided into seven groups randomly, containing ten rats in each group. Citrus peel pectin in different proportions was provided to the rats. The intervention groups of rats were as follows.

G_0_ was taken as a normal control that received a basal diet (no induction).

G_1_ as a negative control, received a basal diet only after disease induction.

G_2_ as a positive control was treated with the standard drug Atorvastatin 10 mg/kg p.o. along with a basal diet after disease induction (El‐Tantawy et al. 2015).

G_3_ was fed with 3% citrus peel pectin after the induction of hyperlipidemia.

G_4_ was fed with 7% citrus peel pectin after the induction of hyperlipidemia.

G_5_ was fed with 11% citrus peel pectin after the induction of hyperlipidemia.

G_6_ was fed with 15% citrus peel pectin after induction of hyperlipidemia.

The dosage of the citrus peel pectin has been adopted from a previous study where rat chow was supplemented with 5% apple‐derived pectin. Therefore, we have selected four percentages to determine the effect at a percentage of < 5%, i.e., 3% and > 5% that include 7%, 11%, and 15% (Jiang et al. 2016).

The body weight, diet, and water intake of rats were measured daily, and the results were recorded. During the 8‐week study period, a normal and citrus peel pectin‐containing diet was provided to the respective groups to distinguish the effect of feed on selected clinical parameters like serum lipid profile, liver functioning, renal functioning, and glutathione level. Citrus peel pectin in different proportions was provided to the rats.

### 2.3 Induction of Hyperlipidemia

Hyperlipidemia was induced by 1.5% cholesterol and 0.5% cholic acid added to the daily normal diet of rats (Bader Ul Ain et al. 2020). The diet was placed in the cage carefully and was administered for 15 days.

### 2.4 Physical Parameters

The physical assessment was performed throughout the whole experiment. Net feed intake and water intake were measured daily. Rat body weight was determined every week and at the end of the experimental period (60 days), respectively, to check the fluctuations and to minimize the effect of bias in the efficacy study.

### 2.5 Biochemical Assessment

Rats were kept for 60 days for a research trial. Blood samples were collected. TC, HDL, LDL, TG, ALT, AST, ALP, blood urea nitrogen, serum creatinine, serum protein, and glutathione content were analyzed.

#### 2.5.1 Collection of Blood

Venous blood was collected in simple and ethylene diamine tetraacetic acid (EDTA) solution‐containing tubes, and these samples were used to analyze the antihyperlipidemic potential of pectin. Rats were anesthetized using chloroform. Cotton soaked in chloroform was placed in a closed container until they lost consciousness (Hamad et al. 2016). Disposable syringes were used to draw blood from the orbital sinus and fill blood sample bottles. Then, the serum was preserved by centrifuging the blood samples at 1500 *g* for 10 min and stored at −80 °C.

#### 2.5.2 Lipid Profile

Serum total cholesterol (TC), triglycerides (TG), high‐density lipoprotein (HDL), and low‐density lipoprotein (LDL) levels were analyzed at the end of the study. The TC, TG, and HDL of the collected serum were measured calorimetrically using Merck's commercial kit, while the serum LDL was calculated by using the formula given in the equation below (Abdelfattah et al. 2024).

LDLCholesterol=TC−HDL−VLDL

VLDL=TG/5

#### 2.5.3 Atherogenic Index

The atherogenic index was estimated by the following equation (Maryanto and Marsono 2019).

$$\text{Atherogenic}\;\text{Index}=\log\left(\frac{\mathrm{TG}}{\mathrm{HDL}}\right)$$

#### 2.5.4 Renal Function Test

The serum creatinine concentration and urea concentration were measured using the autoanalyzer Selectra ProM (Imo et al. 2019). Total serum protein concentration (cat. no. SB‐0250‐500) was determined using a spectrophotometer (5010, Photometer, BM Co, Eiterfeld, Germany) according to the manufacturer's method (Elshopakey and Elazab 2021).

#### 2.5.5 Liver Function Markers

The levels of aspartate aminotransferase (AST), alkaline phosphatase (ALP), and alanine transaminase (ALT) in serum were determined according to the enzymatic colorimetric method using the commercially available diagnostic kit (Eslami et al. 2023).

#### 2.5.6 Antioxidant Status

The antioxidant status was checked by determining the glutathione content (Feng et al. 2011).

### 2.6 Histopathological Studies

A midline thoracotomy was performed, and the heart, liver, and kidneys were removed from the control and experimental animals. Then, specimens of 1 cm thickness from the heart, liver, and kidneys were dissected out, followed by immediate washing with normal saline. After washing, the tissues were placed in appropriate tissue cassettes, and then these cassettes were labeled. The tissues were fixed in a 10% formalin solution (pH 7.2–7.4) for at least 24 h. Subsequently, the fixative was removed under tap water for 2 h. Then, dehydration was performed with 70% ethanol for 2 h and cleared with xylene. After that, the infiltration process was carried out by placing the tissue cassettes in liquid paraffin wax in a hot air oven for 4 h. Afterward, they were embedded in paraffin wax (BMJ‐III embedding machine, Changzhou Electronic Instrument Factory, Jiangsu, China) and then cut into 5 μm thick sections using the slicing instrument called a microtome (Leica RM2235; Leica, Heidelberg, Germany). The tissues were stained with Hematoxylin and Eosin (H&E stain). Then, section mounting was done with the help of mounting medium (gelatin) to preserve and support a section for light microscopy in a water bath at 50°C. The slide was placed at a 45° angle under the surface of water where the tissue film was floating. Subsequently, mount the tissue film over the surface of the slide and label the slide. Before viewing under the microscope, place all mounted slides in a hot air oven for 30 min at 60°C to dry and remove extra paraffin present around the tissue section (Alkhudhayri et al. 2021). Finally, the slides were viewed under the light microscope (Model IM‐910, IRMECO GmbH & CQ, KG Mercatorstr. 62a, 21502 Geesthacht, Germany) with TOUPCAM (Industrial Digital Camera, UCMOS14000KPA, TOUPTEK Photonics Co., LTD) at a magnification of 400×.

### 2.7 Statistical Analysis

Three replicates were performed, and the results of all experiments were expressed as a mean value along with standard deviation. Statistical software Statistix‐8.1 was used for the data analysis. Statistical analysis such as one‐way and two‐way ANOVA, was applied to the study trial data to check the level of significance (Montgomery 2019). Further, the Tukey test was applied as a multiple comparison test.

## 3 Results and Discussion

Dietary interventions owing to their scientific evidence, affordability, and accessibility have gained the attention of patients as well as clinicians regarding disease prevention and health promotion. In this milieu, the current study was conducted to analyze the hypolipidemic effect of citrus peel pectin in rats. Hyperlipidemia induction was confirmed by checking the level of the lipid profile. After the induction of hyperlipidemia, feed intake, water intake, body weight, lipid profile, atherogenic index, liver function test, renal function test, and glutathione level were analyzed, and their results and discussion are described below. The investigated parameters were interpreted statistically to draw valid conclusions.

### 3.1 Feed Intake, Water, and Weight

**FIGURE 1:**
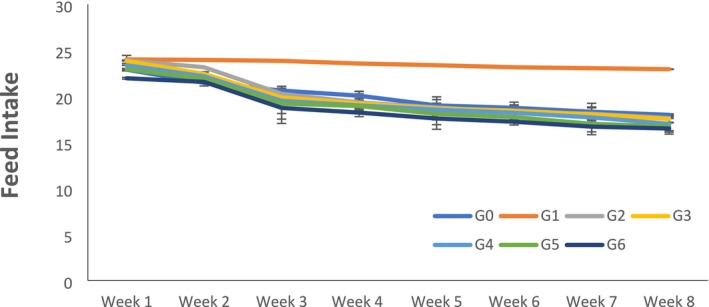
Feed intake of rats according to groups and weeks. Data is expressed as Mean ± SD. Each group contains rats *n* = 10. G_0_ = Normal control (normal feed), G_1_ = Negative control (normal feed, hyperlipidemic), G_2_ = Positive control (normal feed with atorvastatin 10 mg/kg p.o.), G_3_ = Normal feed including 3% citrus peel pectin, G_4_ = Normal feed including 7% citrus peel pectin, G_5_ = Normal feed including 11% citrus peel pectin, G_6_ = Normal feed including 15% citrus peel pectin.

**FIGURE 2:**
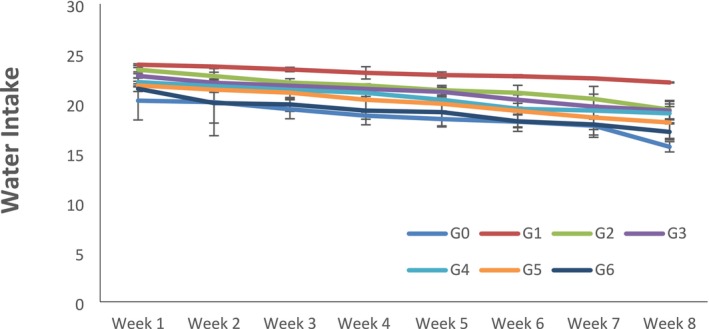
Water intake of rats according to groups and weeks. Data is expressed as Mean ± SD. Each group contains rats *n* = 10. G_0_ = Normal control (normal feed), G_1_ = Negative control (normal feed, hyperlipidemic), G_2_ = Positive control (normal feed with atorvastatin 10 mg/kg p.o.), G_3_ = Normal feed including 3% citrus peel pectin, G_4_ = Normal feed including 7% citrus peel pectin, G_5_ = Normal feed including 11% citrus peel pectin, G_6_ = Normal feed including 15% citrus peel pectin.

**FIGURE 3:**
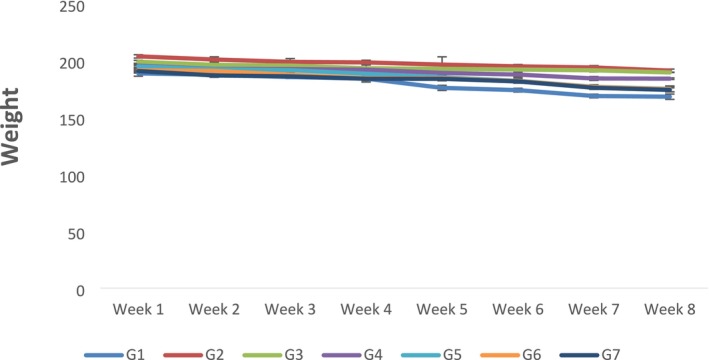
Weight of rats according to groups and weeks. Data is expressed as Mean ± SD. Each group contains rats *n* = 10. G_0_ = Normal control (normal feed), G_1_ = Negative control (normal feed, hyperlipidemic), G_2_ = Positive control (normal feed with atorvastatin 10 mg/kg p.o.), G_3_ = Normal feed including 3% citrus peel pectin, G_4_ = Normal feed including 7% citrus peel pectin, G_5_ = Normal feed including 11% citrus peel pectin, G_6_ = Normal feed including 15% citrus peel pectin.

Statistical inference indicated that the feed and water intake was estimated by measuring the feed and water given and left in the cages and bottles of rats each day. It showed that all the treatments, study week, and interaction effects significantly affected feed intake, water intake, and weight of rats. As the concentration of citrus peel‐derived pectin increased in the diet, feed and water intake were significantly decreased because of the satisfying effect of fiber (Figures 1 and 2). The maximum value for weight was observed in negative control G_1_; on the other hand, body weight significantly decreased in G_6_, where the highest amount (15% of citrus peel pectin) was given to the rats (Figure 3).

It is evidenced from the above inferences of statistical analyses that citrus‐derived peel pectin ameliorates hyperlipidemia in Sprague Dawley rats. Pectin derived from citrus peel is a fiber that could be linked to weight loss. In this study, different concentrations of citrus peel pectin were given to rats. Their body weight was measured every week. Statistical analysis revealed that all the treatments, study weeks, and their interaction effect significantly affected weight. Statistical analysis indicated that, as the amount of pectin increased, the average weight of rats decreased significantly. The findings were in accordance with the study conducted by Zhan et al. (2019), in which pectin reduced the body weight of mice by modulating gut microbiota. The findings of the present study were also in line with the results in which the fermented pectin reduced body weight by inhibiting digestive enzymes (Hamden et al. 2018). Moreover, the results also agreed with those of Kang et al. (2018). Soluble fiber is the main reason behind weight loss. Furthermore, the results are in line with the study on the supplementation of bran in mice suffering from dextran sodium sulfate (DSS) induced colitis, which showed a significant effect on the feed and water intake of mice (Ritchie et al. 2017).

### 3.2 Lipid Profile, Atherogenic Index, and Glutathione Assessment

The highest serum TC, LDL cholesterol, and TG levels were found for the rats in the negative control group consuming cholesterol and cholic acid alone without the intervention of citrus peel pectin compared to baseline values. However, it was well managed by rats consuming 3%, 7%, 11%, and 15% citrus peel pectin after the induction of hyperlipidemia, and the best results were shown by G_6_ consuming 15% citrus peel pectin along with a normal diet. Moreover, HDL concentration increased significantly (*p* ≤ 0.05) in the rats on a diet supplemented with citrus peel pectin (3%, 7%, 11%, and 15%) compared to the negative control group (G_1_) (Table 1).

**TABLE 1:** Effect of different concentrations of citrus peel pectin on lipid profile.

Groups	Serum cholesterol (mg/dL)	LDL (mg/dL)	HDL (mg/dL)	Serum triglycerides (mg/dL)	Atherogenic index	Glutathione (μg/mL)
G_0_	180.67 ± 1.97^bc^	65.18 ± 3.18^f^	60.74 ± 0.46^a^	140.95 ± 0.07^b^	0.36 ± 0.003^e^	164.08 ± 0.26^a^
G_1_	257.07 ± 3.24^a^	155.78 ± 2.47^a^	34.03 ± 0.95^g^	160.44 ± 0.24^a^	0.67 ± 0.01^a^	112.83 ± 3.96^d^
G_2_	181.84 ± 3.31^b^	93.72 ± 2.41^b^	47.57 ± 0.37^f^	135.88 ± 0.07^c^	0.45 ± 0.003^b^	150.73 ± 7.57^c^
G_3_	178.48 ± 1.80^c^	83.80 ± 3.08^c^	49.22 ± 0.39^e^	131.31 ± 0.21^d^	0.42 ± 0.003^c^	154.84 ± 3.54^bc^
G_4_	168.23 ± 2.14^d^	77.02 ± 1.19^d^	52.42 ± 0.43^d^	129.69 ± 0.45^e^	0.39 ± 0.004^d^	157.81 ± 7.01^ab^
G_5_	159.15 ± 2.46^e^	74.25 ± 1.34^de^	54.27 ± 0.32^c^	125.45 ± 0.07^f^	0.36 ± 0.002^e^	161.92 ± 5.73^a^
G_6_	152.10 ± 1.09^f^	72.62 ± 4.66^e^	57.11 ± 0.29^b^	123.95 ± 0.10^g^	0.33 ± 0.002^f^	163.87 ± 2.97^a^

*Note:* Data is expressed as mean ± SD. Each group contains rats *n* = 10. Different letters in the same column represent statistically significant differences between means. G_0_ = Normal control (normal feed), G_1_ = Negative control (normal feed, hyperlipidemic), G_2_ = Positive control (normal feed with atorvastatin 10 mg/kg p.o.), G_3_ = Normal feed including 3% citrus peel pectin, G_4_ = Normal feed including 7% citrus peel pectin, G_5_ = Normal feed including 11% citrus peel pectin, G_6_ = Normal feed including 15% citrus peel pectin.

The atherogenic index significantly increased following the administration of cholesterol and cholic acid in G_1_ compared to the normal control receiving the standard rat diet (G_0_). In contrast, the administration of citrus peel pectin (3%, 7%, 11%, and 15%) significantly (*p* < 0.05) decreased the atherogenic index in G_2_, G_3_, G_4_, G_5_, and G_6_, respectively. The lowest atherogenic index was noticed in G_6_ (15% citrus peel pectin), and the highest atherogenic index was found in G_1_ (negative control) (Table 1). The mean concentrations for serum glutathione showed significant differences (*p* ≤ 0.05) among the various rat groups. The lowest value of glutathione (112.83 μg/mL) was observed in the negative control group, followed by G_2_, G_3_, G_4_, G_5_, and G_6_, showing resistance against the increase in oxidative stress (Table 1).

By increasing the concentration of citrus peel pectin, the value of serum TC and LDL cholesterol decreased while HDL cholesterol increased as mentioned in a study conducted by (Zhu et al. 2017). Similarly, the results coincided with another study's findings, where different concentrations of corn flour altered the serum lipid profile (TC, LDL, TG) while increasing the HDL level in albino mice (Iqbal et al. 2022). The findings of this study showed that haw pectin Penta‐oligogalacturonide reduced the serum TC, TG levels, and LDL by promoting the biosynthesis of bile acid and its excretion in feces. The findings also reported similar results, which showed that pectin decreased the serum TC, TG, and LDL through gut microbiota modulation (Zhan et al. 2019). Moreover, the findings also corroborated a previous study (Kang et al. 2018). The water retention ability of soluble dietary fiber increases the bulk weight and dilution of nutrients within the gastrointestinal tract owing to the presence of water inside carbohydrates and lipids and their course of action across the intestinal wall (Dai and Chau 2017). These properties of soluble dietary fiber, including its capacity to increase satiety and reduce food intake, have been recognized as important mechanisms for lowering lipid levels (Nie and Luo 2021). Furthermore, this reduction can be attributed to their ability to bind to bile acids/salts, hindering their reabsorption into the enterohepatic circulation and helping their excretion in feces. Subsequently, this process prompts the formation of new bile salts from cholesterol, ultimately leading to a lipid‐lowering effect (Bakr and Farag 2023; Shirouchi et al. 2011). Besides, the upregulation of hepatic LDL receptors may be responsible for the decreased serum LDL concentration by restoring the cholesterol reserves in the liver (Shirouchi et al. 2011). Moreover, a mechanism involves the decrease of the postprandial blood glucose, which reduces insulin production. This might lead to a reduction of cholesterol biosynthesis, as insulin stimulates 3‐hydroxy 3‐methylglutaryl coenzyme A reductase. Finally, cholesterol synthesis in the liver might be inhibited through the fermentation of soluble dietary fiber by the intestinal microbial flora into short‐chain fatty acids, i.e., acetate, propionate, or butyrate (Gunness and Gidley 2010). The propionate and acetate are readily absorbed, while butyrate is broken down by the mucosal cells of the colon. The short‐chain fatty acids production and, in particular, the changes in the ratio of propionate and acetate may affect lipid metabolism, reducing hepatic absorption and increasing cholesterol excretion via bile and fecal lipids (Wong et al. 2006). Another hypothesis for the anti‐hypercholesterolemic effects of soluble dietary fiber is based on a lower overall energy intake. The fiber‐rich foods contain fewer calories and take longer to digest, consequently promoting satiety (Surampudi et al. 2016).

The severity of dyslipidemia, such as high levels of TG and low HDL associated with a small size of LDL particles, governs a high atherogenic index and consequently atherogenic risk (Capomolla et al. 2019). These results indicated that citrus peel pectin could reduce the risk of atherosclerosis caused by the intake of a diet enriched with cholesterol (1.5%) and cholic acid (0.5%). Interestingly, the reduction of the atherogenic index is primarily the decrease of lipoprotein rate, which is proatherogenic (triglycerides) and LDL, and an increase in HDL (anti‐atherogenic). The current research findings were consistent with the results of Zhu et al. (2015), where haw pectin pentasaccharide decreased the atherogenic index from 5.85 to 3.47. Furthermore, Hamauzu et al. (2011) reported that dried onion powder reduced the atherogenic index of rats over 18 weeks. In dried onion powder, large amounts of quercetin and pectin might contribute to the anti‐atherogenic effect. The red guava and pectin treatment decreased the atherogenic index from 0.40 to 0.05 (Maryanto and Marsono 2019). The beverage supplemented with black chokeberry extract enriched with 1% pectin significantly improved the atherogenic index (LDL/HDL) in aging rats (Daskalova et al. 2021). The data from Capomolla et al. (2019) indicated that both low‐ and high‐dose pectin‐enriched bergamot polyphenol extract complex decreased the mean value of the atherogenic index to 0.27 and 0.19. The supplementation of polyphenol and pectin‐enriched golden kiwifruit lowered the atherogenic index of rats to 1.29 as compared to rats only fed with a high‐fat diet (Alim et al. 2023).

By neutralizing the reactive oxygen species, glutathione plays an important antioxidant role, as it is the most abundant thiol in mammalian cells (Rakha et al. 2023). Moreover, the increase in glutathione value in the present study is indicative that the citrus peel pectin has antioxidants that have good bioavailability in the body (Liu et al. 2022). The results align with earlier studies' findings (AboZaid et al. 2018; Amr 2011).

These outcomes specified that citrus peel pectin might have the potential to alleviate dyslipidemia by improving serum lipid homeostasis, atherogenic index, and antioxidant status in cholesterol and cholic acid‐fed rats.

### 3.3 Liver Function Test

Statistical inferences indicate that all the treatments given to rats significantly affected the levels of ALT, AST, and ALP (Table 2). As the citrus peel pectin supplementation increased, the serum ALT, AST, and ALP levels decreased significantly. Negative control (G_1_) had a high level of serum ALT, AST, and ALP, and the lowest level was observed in (G_6_) with the highest level of citrus peel pectin supplementation (15%) followed by G_5_, G_4_, G_3_, and G_2_. The findings agreed with a study that showed a decline in serum ALT levels (Amr 2011). Furthermore, the results coincided with a previous work, which exhibited a significant reduction in serum AST and ALP in hyperlipidemic rats fed with Ortanique peel polymethoxylated flavone extract (Green et al. 2013). The supplementation of citrus peel pectin powder displayed resistance against any detrimental variations in liver function, showcasing its hepatoprotective capacity and potentially acting as a functional remedy for liver diseases. The effect is attributed to its antioxidant potential, which provides support against oxidative damage brought on by reactive oxygen species (Liu et al. 2022).

**TABLE 2:** Effect of different concentrations of citrus peel pectin on ALP, AST, and ALT levels.

Groups	ALP (IU/L)	AST (IU/L)	ALT (IU/L)
G_0_	93.56 ± 5.17^e^	32.52 ± 2.73^c^	38.49 ± 2.75^b^
G_1_	150.43 ± 3.36^a^	59.44 ± 4.24^a^	66.49 ± 3.76^a^
G_2_	141.63 ± 3.70^b^	36.70 ± 1.81^b^	32.95 ± 2.91^cd^
G_3_	134.05 ± 2.60^c^	26.53 ± 2.19^d^	36.01 ± 2.29^bc^
G_4_	109.01 ± 2.23^d^	21.60 ± 1.50^e^	32.05 ± 2.55^d^
G_5_	95.18 ± 1.91^e^	14.41 ± 1.95^f^	19.97 ± 1.76^e^
G_6_	90.95 ± 1.25^e^	14.06 ± 1.73^f^	19.46 ± 1.59^e^

*Note:* Data is expressed as mean ± SD. Each group contains rats *n* = 10. Different letters in the same column represent statistically significant differences between means. G_0_ = Normal control, G_1_ = Negative control (normal feed, hyperlipidemic), G_2_ = Positive control (normal feed with atorvastatin 10 mg/kg p.o.), G_3_ = Normal feed including 3% citrus peel pectin, G_4_ = Normal feed including 7% citrus peel pectin, G_5_ = Normal feed including 11% citrus peel pectin, G_6_ = Normal feed including 15% citrus peel pectin.

### 3.4 Renal Function Test

Findings for serum urea are shown in Table 3, indicating that different concentrations of pectin had a highly significant effect on blood urea nitrogen and serum creatinine levels. In contrast to a negative control (G_1_), the lowest level was observed in G_6_, with the highest level of citrus peel pectin supplementation followed by G_5_, G_4_, G_3_, and G_2_. Thus, it is indicated that citrus peel pectin at the concentration of 15% showed no negative effects or toxicity.

**TABLE 3:** Effect of different concentrations of citrus peel pectin on blood urea nitrogen, serum creatinine, and serum protein.

Groups	Blood urea nitrogen (mg/dL)	Creatinine (mg/dL)	Serum protein (g/dL)
G_0_	17.45 ± 0.11^c^	0.87 ± 0.06^b^	6.68 ± 0.45^bc^
G_1_	23.00 ± 0.28^a^	2.59 ± 0.39^a^	9.01 ± 0.49^a^
G_2_	19.23 ± 0.28^b^	0.84 ± 0.05^bc^	6.64 ± 0.59^bc^
G_3_	16.77 ± 0.10^d^	0.77 ± 0.03^bc^	6.79 ± 0.57^b^
G_4_	15.72 ± 0.06^e^	0.75 ± 0.04^bc^	6.39 ± 0.43^bc^
G_5_	13.47 ± 0.07^f^	0.67 ± 0.06^bc^	6.25 ± 0.38^bc^
G_6_	12.78 ± 0.09^g^	0.62 ± 0.07^c^	6.09 ± 0.52^c^

*Note:* Data is expressed as Mean ± SD. Each group contains rats *n* = 10. Different letters in the same column represent statistically significant differences between means. G_0_ = Normal control (normal feed), G_1_ = Negative control (normal feed, hyperlipidemic), G_2_ = Positive control (normal feed with atorvastatin 10 mg/kg p.o.), G_3_ = Normal feed including 3% citrus peel pectin, G_4_ = Normal feed including 7% citrus peel pectin, G_5_ = Normal feed including 11% citrus peel pectin, G_6_ = Normal feed including 15% citrus peel pectin.

Statistical inference for serum protein indicated that different concentrations of pectin significantly affected serum protein (Table 3). The hyperlipidemic rats have exhibited a highly significant result upon being fed different citrus peel‐derived pectin doses. As the level of citrus peel pectin increased, the serum protein level decreased significantly. As compared to the negative control (G_1_), the lowest value for serum protein was observed in G_6_, with the highest level of citrus peel pectin supplementation followed by G_5_, G_4_, G_3_, and G_2_.

As the addition of citrus peel pectin increased, the blood urea nitrogen and serum creatinine levels decreased significantly, as demonstrated by the findings in which blood urea nitrogen and serum creatinine levels were decreased by the administration of essential oil of citrus in rats (Bouzenna et al. 2016). The findings of this study were also concurred with by AboZaid et al. (2018). Moreover, the results agreed with the findings of Ehsan et al. (2023). The findings coincided with the study of Bouzenna et al. (2016), in which serum protein levels significantly decreased by administering essential citrus oil in rats.

### 3.5 Histopathological Responses of Heart Tissues

**FIGURE 4:**
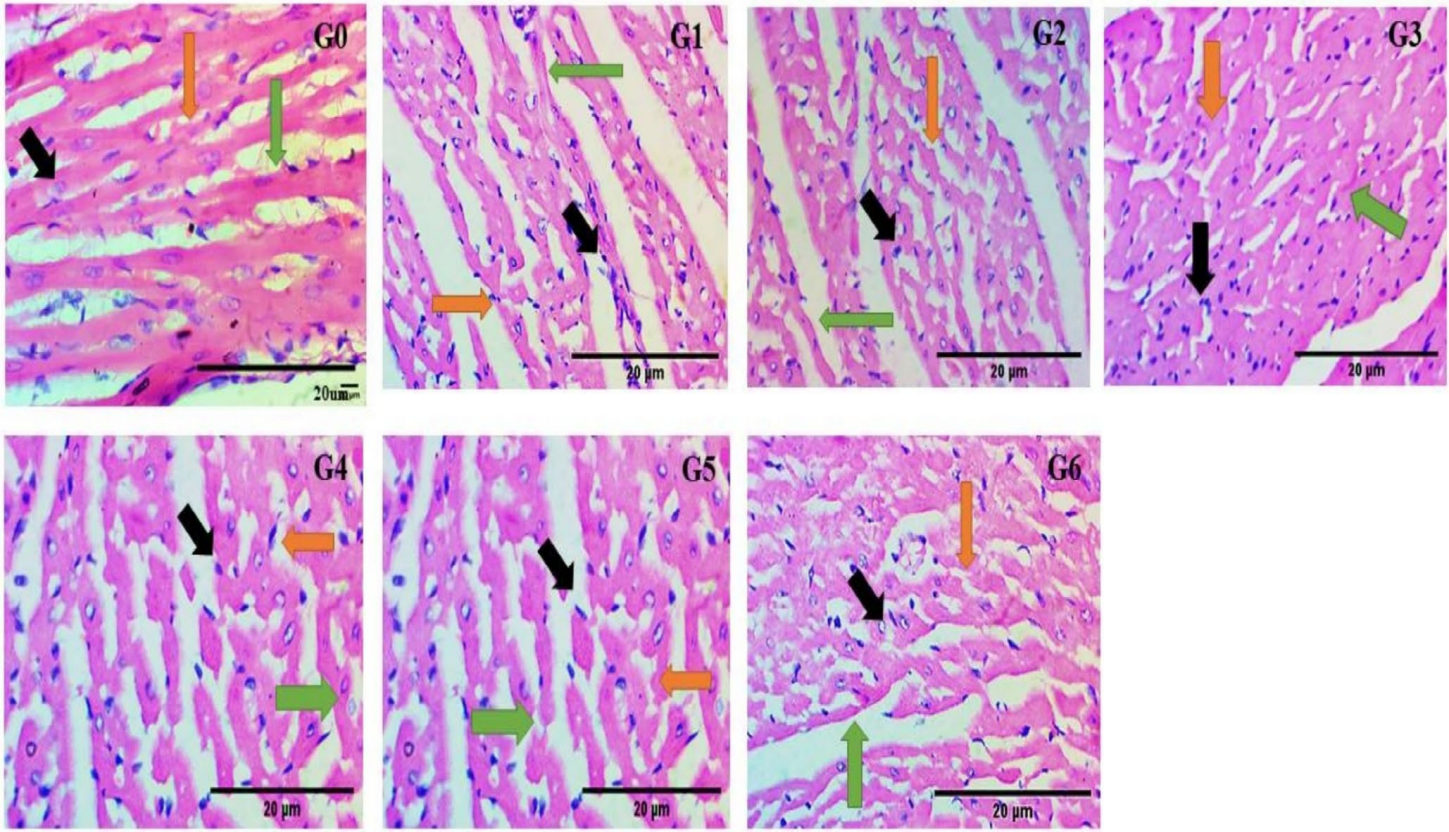
Effect of histopathological changes of the heart in control and experimental animals under 400×. G_0_ = Normal control (normal feed), G_1_ = Negative control (normal feed, hyperlipidemic), G_2_ = Positive control (normal feed with atorvastatin 10 mg/kg p.o.), G_3_ = Normal feed including 3% citrus peel pectin, G_4_ = Normal feed including 7% citrus peel pectin, G_5_ = Normal feed including 11% citrus peel pectin, G_6_ = Normal feed including 15% citrus peel pectin.

Microscopic insights of the heart tissue captured at 40× (G_0_) showed the normal architecture and structural organization of the heart tissue (as black arrows for muscle cell nuclei, green arrows for the intercalated disc with demarcated striations, and orange arrows for the specialized branches of the heart muscles) (Figure 4). G_1_ showed marked deviations in the normal histological structures as hypertrophy of the muscle cells' nuclei located peripherally, depletion of the intercalated disc with lack of striations, increase in the interstitial spaces, and less specialized branches of the heart muscles. In G_2_ there was a regaining of the branched system and an organized intercalated disc toward normal muscle cells. In G_3_, G_4_, G_5_, and G_6_ showed mild to moderate restoration of the specialized branch system, striations appearance, decreases in the interstitial space, and decreases in the number of cells with centrally located nuclei.

### 3.6 Histopathological Responses of Kidney Tissues

**FIGURE 5:**
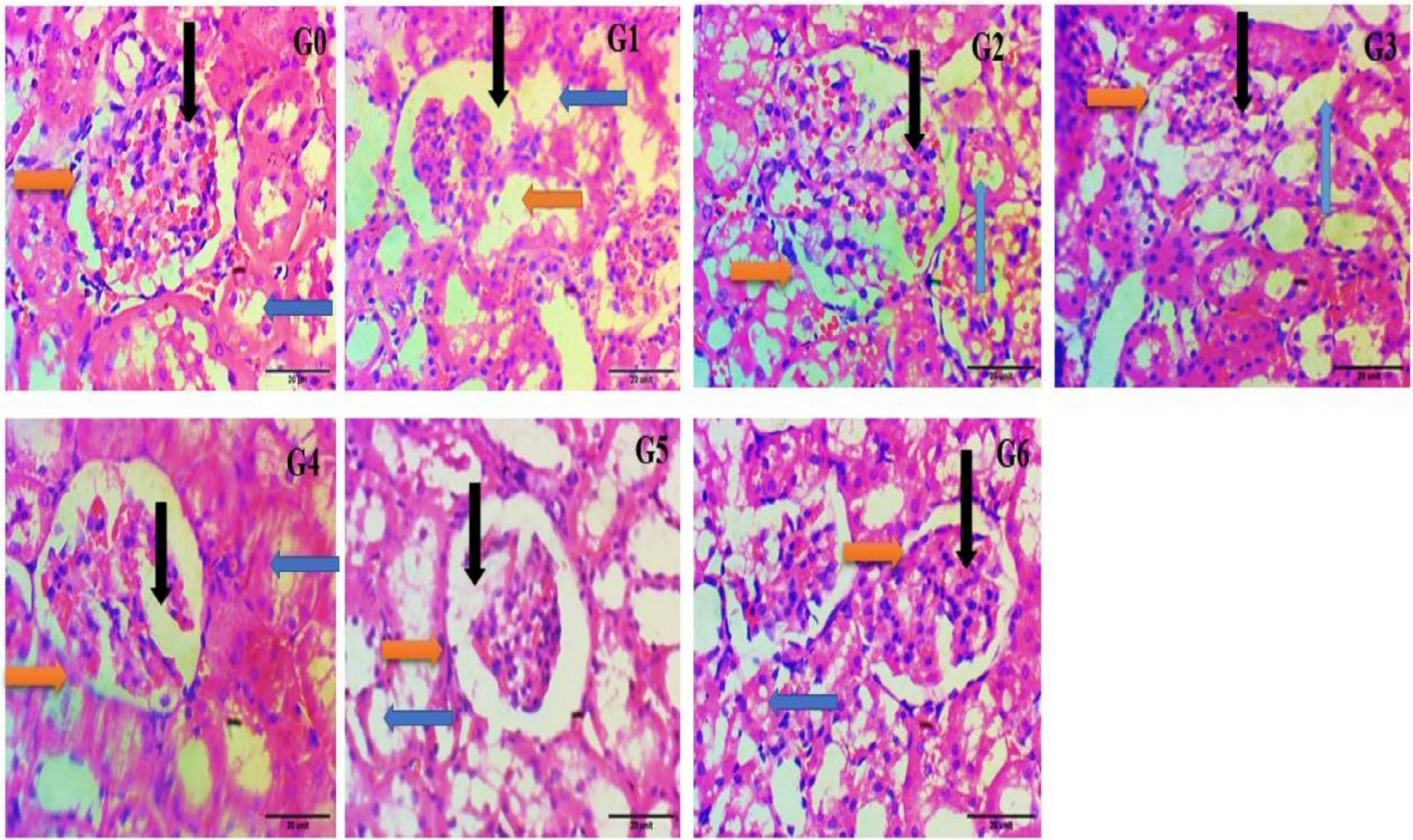
Effect of histopathological changes of the kidney in control and experimental animals under 400×. G_0_ = Normal control (normal feed), G_1_ = Negative control (normal feed, hyperlipidemic), G_2_ = Positive control (normal feed with atorvastatin 10 mg/kg p.o.), G_3_ = Normal feed including 3% citrus peel pectin, G_4_ = Normal feed including 7% citrus peel pectin, G_5_ = Normal feed including 11% citrus peel pectin, G_6_ = Normal feed including 15% citrus peel pectin.

Histology of the kidney tissues captured at 40× showed that the G_0_ has normal parenchyma of the kidney tissue with intact histological structures (blue arrows depict the proximal renal tubules, black arrows represent the glomerulus having mesangial cells in it, while orange arrows exhibit the Bowman's capsule and capsular space) (Figure 5). G_1_ showed the prominent changes in the structures like disoriented Bowman's capsule, increase in capsular space, loss of glomerular portion with decrease in the number of mesangial cells, and loss of renal proximal tubular system. In G_2_, G_3_, G_4_, G_5_, and G_6_, there were mild to moderate reversions of the glomerular structures with regaining of the mesangial cells, decreases in the capsular space, and well‐organized Bowman's capsule, as shown in Figure 5.

### 3.7 Histopathological Responses of Liver Tissues

**FIGURE 6:**
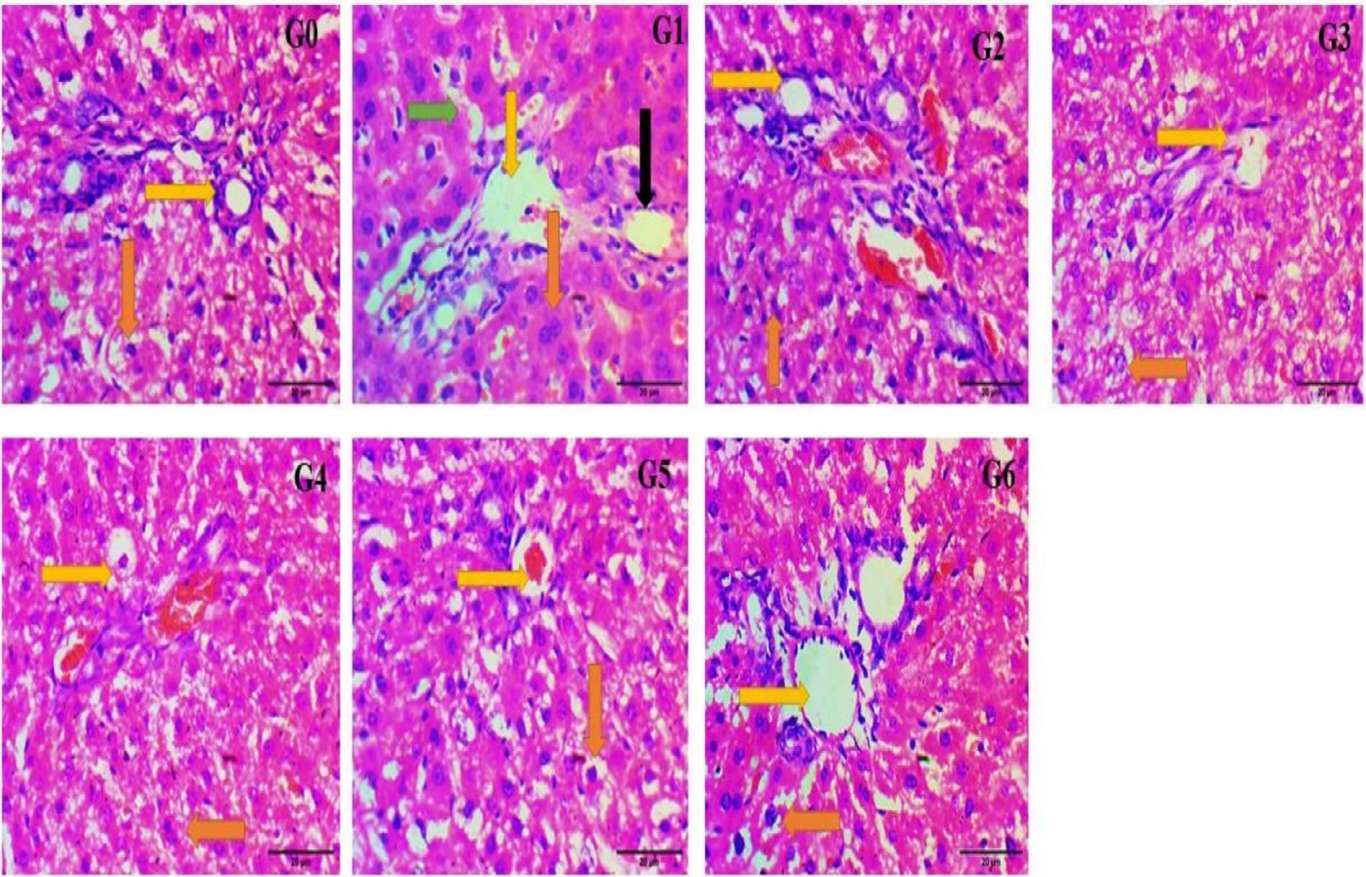
Effect of histopathological changes of liver in control and experimental animals under 400×. G_0_ = Normal control (normal feed), G_1_ = Negative control (normal feed, hyperlipidemic), G_2_ = Positive control (normal feed with atorvastatin 10 mg/kg p.o.), G_3_ = Normal feed including 3% citrus peel pectin, G_4_ = Normal feed including 7% citrus peel pectin, G_5_ = Normal feed including 11% citrus peel pectin, G_6_ = Normal feed including 15% citrus peel pectin.

Microscopic examination of the liver tissue of G_0_ at 40× showed normal features of the liver tissues with a central vein, bile duct, hepatic artery, sinusoids, and a centralized nucleus in the hepatocytes. Yellow arrows depict the central vein, orange arrows exhibit the hepatocytes with nuclei, green arrows represent the sinusoids, and black arrows show the vacuolization and accumulation of the fat. In G_1_, there were well‐defined changes that had been observed with enlargement and disorientation of the central vein, vacuolization in the cytoplasm, hypertrophy of the hepatocytes (nucleus toward the periphery), and enlargement of the sinusoids. Whereas in G_2_, G_3_, G_4_, G_5_, and G_6_, there was a maintaining and reversion toward the normal parenchyma from mild to moderate with the removal of the vacuolization, shortening of the sinusoids, and hepatocytes with a centralized nucleus as shown in Figure 6.

From the histopathological analysis, it has been seen that low to moderate and moderate to high impact on the histology of the heart, liver, and kidney tissues due to different concentrations of the pectin. The amelioration of the histopathological changes was quite similar to that observed by the *Ocimum sanctum* L. leaves extract in the myocardial cells of rats fed a high‐cholesterol diet (Suanarunsawat et al. 2011). Similarly, Hassanein et al. (2015) studied that 10% and 20% of carob powder supplementation decreased the hemorrhage, mononuclear cell infiltration, and necrosis of myocardial muscles produced by a hyperlipidemic diet containing 10% animal fat and 1% cholesterol. Therefore, the present study clearly shows that citrus peel pectin may be of therapeutic importance, not only as a lipid‐lowering agent in serum but also as a cytoprotective agent to protect the cardiomyocyte's injury from hyperlipidemia as well as liver and kidney cells.

## 4 Conclusion

Hyperlipidemia is a serious public health concern that extensively contributes to the occurrence of atherosclerosis, which is one of the factors that trigger coronary heart diseases, but may be improved through modifications in dietary patterns. The current study aimed to determine the lipid profile and atherogenic index of Sprague Dawley hyperlipidemic rats, which were given citrus peel pectin in different ratios of 3%, 7%, 11%, and 15%. The findings demonstrated that pectin significantly reduced the levels of TC, TG, and LDL while increasing the HDL levels. These changes in the lipid profile led to a decreased atherogenic index; consequently, the risk of atherogenesis decreased. Furthermore, it might have prevented the initiation of atherosclerotic lesions in the heart and significantly mitigated the changes caused by hyperlipidemia. Further, immune cell infiltration was not very conspicuous in the heart of rats fed with citrus peel pectin (15%), and it might be due to its potent anti‐angiogenic effect, which needs to be explored further. The current study is unique in that it uses citrus peel pectin, a readily available and frequently discarded by‐product of the fruit industry, to treat hyperlipidemia. In contrast to earlier research that focused on whole‐fruit pectin or citrus peel powders, this study highlights an economical and sustainable way to value citrus peel pectin as a useful dietary ingredient. Through the use of in vivo models, the study offers new information about the lipid‐lowering capabilities of citrus peel pectin, adding to the increasing amount of data that supports polysaccharides as naturally occurring hypolipidemic agents. The findings of the study suggest several new avenues for research. Future studies should focus on elucidating the specific molecular mechanisms through which citrus peel pectin influences lipid metabolism. To establish its safety and efficacy for therapeutic use, clinical trials involving human subjects are required. Furthermore, exploring potential synergistic effects with other bioactive compounds or dietary interventions could enhance the hypolipidemic properties of citrus peel pectin. Advances in nutraceutical formulation and food product development may also enable the incorporation of citrus peel pectin into functional foods aimed at addressing metabolic disorders. Moreover, this novel component must be explored against atherosclerosis and non‐alcoholic fatty liver disease through molecular targets and gene expression pathways. The effect of citrus peel pectin as a prebiotic on gut microbiota must also be explored. Further research is needed to explore citrus peel pectin as a natural dietary supplement for controlling hyperlipidemia. Based on the outcome of current studies, it is recommended that food industries view citrus fruit peels as a significant source of functional ingredients instead of a by‐product. The citrus peel pectin must be developed into a nutraceutical formulation just like the sachet of psyllium husk. Moreover, public health initiatives promote the incorporation of fiber‐rich by‐products such as citrus peel into regular diets. Future research should utilize larger sample sizes and extended intervention durations to thoroughly evaluate the long‐term impacts of citrus peel pectin.

## 5 Limitations

Although the current study presents promising results, it is important to recognize several limitations. While the Sprague Dawley rat model is useful for preclinical evaluation, it may not fully capture the intricacies of human lipid metabolism, highlighting the need for further validation through clinical trials. Moreover, the two‐month intervention duration, while adequate for detecting initial changes, may not truly represent the long‐term effectiveness and safety of citrus peel pectin in addressing chronic hyperlipidemia. This research primarily concentrated on biochemical parameters and histopathological examinations without delving into the molecular mechanisms or gene expression pathways that could explain the observed effects. Additionally, since only glutathione was assessed as an antioxidant biomarker, it restricts a more comprehensive understanding of the oxidative stress profile. The lack of analysis on gut microbiota also limits insights into the possible prebiotic effects of pectin. Finally, the study did not investigate the inclusion of citrus peel pectin in functional food formulations, which might increase its applicability in public health nutrition.

## Author Contributions

**Hina Rasheed:** data curation (equal), Formal analysis (equal), investigation (equal), writing – original draft (equal). **Ammar B. Altemimi:** writing – review and editing (equal). **Roshina Rabail:** visualization (equal), writing – original draft (equal). **Sidra Tul Muntaha:** writing – original draft (equal), writing – review and editing (equal). **Allah Rakha:** data curation (equal), formal analysis (equal), investigation (equal), methodology (equal). **Usman Haider:** validation (equal), writing – original draft (equal). **Fareha Rasheed:** writing – review and editing (equal). **Amin Mousavi Khaneghah:** data curation (equal), writing – review and editing (equal). **Gholamreza Abdi:** conceptualization (equal), software (equal), visualization (equal), writing – review and editing (equal), funding. **Rana Muhammad Aadil:** data curation (equal), formal analysis (equal), investigation (equal), project administration (equal), writing – review and editing (equal), Supervision.

## Ethics Statement

The study involves animal testing. Therefore, it was approved by the Institutional Animal Care and Use Committee (IACUC) (vide No. 5285/ORIC, dated 28‐8‐2024), University of Agriculture, Faisalabad, Pakistan.

## Conflicts of Interest

The authors declare no conflicts of interest.

## Data Availability

Instead of being uploaded to a website, the data is stored on my desktop computer so that I can send you the data at your request.
